
JOURNAL INFORMATION
==============================
NLM Title Abbreviation: Med Chir Rev
NLM Journal ID: 0034617

The Medico-Chirurgical Review

Publisher: Biomedical Journal Digitization Project

ARTICLE INFORMATION
==============================
PMCID: PMC4966248
PMID: 29917620
Article version: 1
Subjects: Articles

Hospital Practice

Print publication date: 1828 Oct 1
Volume: 9
Issue: 18
First page: 481
Last page: 490
1828 Aug
License: This work is free of known copyright restrictions. For more information, please see PMC Back Issue Digitization.
License URL: https://creativecommons.org/publicdomain/mark/1.0/

==============================

### 38. REMARKABLE CASE OF NEURALGIA. By MR. DOD.

Among the numerous suggestions that have been transmitted to us respecting the distressing case of neuralgia, published in the present Number of this Journal, there is a case related by Mr. Dod, late of Bath, and now of Caroline Street, Bedford Square, which we deem it a duty to communicate to the public, and that in the words of the communicant.

"The distressing case of neuralgia inserted for consultation in a recent fasciculus of the Medico-Chirurgical Review, induces me to communicate some particulars of a most inveterate case of this complaint, which occurred in the person of an officer, who had been most dreadfully lacerated by a tiger in the East Indies.

"The patient was a Lieutenant Saunders, (now Captain) of the East India Company's Service, about 38 or 40 years of age. This gentleman, after having resided in India about eight or nine years, was recommended to return to England on account of a liver-complaint, the sequela of a *jungle fever* he caught during some military operations in which he had been engaged in the country. After a short residence in England, his health being restored, he again returned to India. Upon his return, and during some part of the Poona war, in which he was conspicuously engaged, he one day incautiously approached a tiger, which sprang at him, seized him, and lacerated him in a most dreadful manner, destroying with its claws almost all the pectoral muscle, and other motors of the humerus, of the left arm. The tiger having left him, he lay in this mangled condition for a considerable time before assistance could be procured, during which interval he bled profusely. He recovered from his wounds; but there supervened one of the most distressing neuralgic affections of the body that can well be imagined. He became afflicted with violent head-achs, which increased to such a degree of severity, as sometimes to deprive him of reason. Coupled with these distressing head-achs, there was great pain in the lacerated parts, with subsultus of the muscles and tendons, which frequently extended to those of the body, giving to the spectator the idea or appearance of its being an epileptic fit with which the patient had been seized. His digestive organs were much disordered, and his food in general disagreed with him. When his stomach was most out of order, all his sufferings were exasperated. These distressing complaints continued to increase in severity from the time of the accident until I saw him, about two years ago, at TRURO, to the neighbourhood of which place he had retired to pass the remainder of his miserable life, after having sought relief from some of the most eminent of the faculty in London and other parts of this country. When I saw Mr. S. he exhibited a most distressing spectacle;—His body was emaciated—his countenance was pale—his lips blanched—and his features shrunk. The functions of his digestive organs were much disordered. His extremities were, in general, cold; and his pulse, which was small, beat about 120 strokes in a minute. When paying my second visit to him, I had an opportunity of witnessing one of those nervous seizures above-mentioned, and of noting its progress. The subsultus commenced in the muscles of the lacerated arm; then extended to the eyes and muscles of the face; and, lastly, over the whole body. Some of the muscles seemed in a spastic state, whilst others were in a state of subsultus. He did not struggle! In this condition he remained about ten minutes, and then gradually recovered. On the preceding night, he had suffered severe pain in the lacerated parts, with violent head-ach; the latter symptom continued after the seizure. Although the subsultus frequently took place in the lacerated parts, yet it did not always extend to the body.

"I prescribed the following medicines, which had proved successful in a severe case of neuralgia a few years before.

"℞. Extract. colocynth. comp.—extr. jalap.—extr. alois spicat. āā ℈ij. Mass. pilul. hydrarg. ℈j. Contunde et divide in pilulas xxx æquales.

"Capiat decoct. sarsapar. simp. ℔j. quotidie.

"Balneum calidum 3tio vel 2ndo die.

"I ordered him to take one, two, or three of the pills *every night going to bed,* so as to produce, the next day, two or three evacuations from the bowels. The sarsaparilla he took regularly every day, but the warm bath was not attended to so regularly as I could have wished.

"These were the *only* medicines and means made use of, until my patient perfectly recovered, which he did in *twelve weeks* from the time I first saw him. He embarked the following spring for India, and has continued in good health up to the last accounts I heard of him."

---

### 39. MEDICAL DOCTRINES.

M. Cayol, who recently succeeded M. Chomel in the medical CLINIQUE of LA CHARITÉ, has been amusing the Parisian students excessively by the *novelty* of the doctrines which he has broached. He bewails, in strong terms, the infatuation of his countrymen, who have been led away from the Hippocratic observation of Nature and of symptoms by the ignis fatuus of pathological anatomy! He anathematises those narrow-minded theories which can see nothing but IRRITATION, that fantastic and perverse being, pervading our various organs, and producing all diseases. He conjures the students to seek for nothing more in morbid anatomy than a knowledge of the means by which diseases have ultimately triumphed over nature and art. He tells them, and tells them truly, that they need not expect to discover the *essence* of maladies in the changes of structure which these maladies leave in the dead body.

M. Cayol has enunciated some ideas respecting acute diseases, which are by no means chimerical—many of them, indeed, are such as the most philosophic physicians still entertain.

He observes that nothing is more curious than the great number of characters or phenomena which are *common* to acute diseases in general. It is from these common characters that we are to draw our indications of cure—not from the local organic changes which only take place at certain epochs of the original disease. Look at pneumonia, pleurisy, fever—indeed at the majority of acute diseases, and trace their causes. You will find, what Hippocrates long ago observed, that they are generally attributable to noxious atmospheric impressions, that have disturbed the functions of our organs. But this is not the most remarkable circumstance respecting acute diseases. If we watch their invasion, development, progress, form, and seat, even their termination, we shall find but one continuous struggle of Nature against a morbific cause.

First, we have more or less of horripilation, chilliness, malaise, oppression, recoil of the fluids from the surface to the interior, all which denote the impression of a morbific principle. Next, we have restlessness, elevation of the pulse, heat, &c.—in short, the phenomena of *re-action*, which is neither more nor less than an effort of Nature to drive this morbific principle or cause to some point or other of the body. It is then, *and then only*, that the weakest organ becomes the local habitation or *seat* of the malady. Thus, then, we see, contrary to the prevailing opinion of the present day, that all *local* affections are first *general* affections, and that their localization is the result of Nature's effort to disembarrass herself of an enemy that threatens life. Thus a pneumonia, for example, is only the *crisis*, that is, the misplaced or unfortunate *localization* of a general disorder, resulting from a morbific impression on the system.* From these premises it follows, that the physician should be cautious how he interferes rashly during the period of invasion—and even during re-action. "His office, in fact, does not fully commence till the moment when the *morbid localization* becomes established." Till then he should, at most, only aid Nature in the way which she may indicate. When the local malady has manifested itself, the physician should be aware that Nature is still the grand agent, and that her efforts should not be rudely crossed or counteracted. On the contrary, he should di-

* There is much foundation for this assertion. Thus a man is exposed to wet and cold on the top of a coach, and next day he begins to feel all the phenomena of invasion, as above described. Re-action takes place, and, in short, the symptoms of fever are observed *before* there is any affection of the lungs. Is it reasonable, is it philosophic, to call this fever the mere symptomatic fever resulting from the local inflammation in the lungs—a topical affection that shews itself posterior to the phenomena of general fever? We think not.

ligently watch her movements, and endeavour to facilitate her operations, rather than attempt to take the work entirely out of her hands, as too many "bold practitioners" do, to the cost of the patient!

In these days of heroic remedies, the foregoing lecture from a disciple of Hippocrates may not be entirely useless.

---

40. Case of Disease of the Heart. By J. H. Wishart, F.R.S.E.

[Ed. Med. Chir. Trans. Vol. III.]

A married woman, aged 28, came under the author's observations on the 28th March, 1828, affected with palpitation of the heart of several years' duration.

"The pulsation is very irregular and intermitting; it is not confined to the natural situation between the sixth and seventh ribs, but it may be felt nearly as high as the clavicle on the left side of the sternum. The beat is so strong, that it is often heard by her attendants; and, on approaching the ear, the sound is very loud. She has a constant, severe tickling cough, with great anxiety and hurried breathing; the jugular veins have a distinct undulatory motion. The pulse at the wrist is so feeble and irregular, that it can scarcely be numbered. Her chest is very much emaciated, and the mammæ shrunk. The lower extremities are œdematous; the swelling extends up to the pelvis, and there appears also to be slight effusion in the abdomen, but no fluctuation. Urine very scanty and high coloured; bowels open; appetite natural; considerable thirst." The complaint had lasted about seven years, during which she has been unable to walk quick or go up stairs without inducing violent palpitation. Some aperient and diuretic medicines were prescribed, as she would not submit to bleeding. In a few days the whole body became tinged of a yellow colour. There was no enlargement of the liver. There was, from this time, a rapid aggravation of the symptoms. The œdema of the extremities increased—the surface became shining—"and the yellowness of the whole body increased to a greater pitch than I had ever seen it." She expired on the 18th April.

*Dissection.* On opening the thorax, the pericardium and heart were found to occupy the space between the second and eighth ribs, but more inclined towards the right side than usual. The lungs were healthy—twelve ounces of serum in the pleural cavities—six ounces in the pericardium. The heart was tinged of a yellow colour, and was rather enlarged in size. There was a large polypous concretion in the right ventricle, extending into the auricle, and firmly adhering to the columnæ carneæ. The parietes of the left ventricle were slightly hypertrophied. The mitral valves were cartilaginous, and the orifice so contracted, as scarcely to admit the point of the little finger. The opening of the aorta appeared quite closed by a caruncular excrescence, with fringed edges, which projected into the ventricle. The left auricle was much distended, and filled with a fleshy concretion, mixed with blood. The venæ cavæ were larger than natural. There was some dark-coloured serum in the abdomen—right lobe of the liver enlarged, and its surface covered with white tubercles, of the size of peas—similar tubercles were found throughout the substance of the organ—gall-bladder collapsed—ductus communis impervious.

The pulsation of the jugular veins, in this case, is ascribed by Mr. Wishart to the polypous concretion found in the right ventricle. The great action of the heart, as compared with the feeble pulse at the wrists, is satisfactorily accounted for by the state of the semilunar valves of the aorta. In the above case, there must, of course, have been a regurgitation of blood from the left ventricle into the left auricle, as the auriculo-ventricular opening could not be closed by the indurated mitral valve. This caused the whizzing sound which was heard. But this regurgitation acted also on the pulmonary veins, in which (could they have been seen) there would have been found a retrograde pulsation, the same as in the jugulars. This accounts for the cough and dyspnœa, the circulation through the lungs being greatly retarded. It is unusual to find so much valvular disease at the age of 28 years. The closure of the ductus communis hepaticus is not accounted for. We believe, from what we see and read, that diseases of the heart are still on the increase! We attributed their frequency, thirty years ago to "the storms of the revolution." Now we have only the "piping times of peace" to accuse. But, alas! the heart of man, and of woman too, is much more frequently ruffled by the breeze of domestic trouble, than by hurricanes of political events. Even in those fearful "storms of the revolution," it was the *domestic* affliction that broke the human heart.

---

41. BREATHING OF COOL AIR IN PULMONARY DISEASE.

Dr. Drake of New York, in a letter to Dr. Smith, of Philadelphia, has offered some curious speculations as well as results of experience in the treatment of pulmonary diseases. The plan which Dr. D. employs, and his reasons for the adoption of it will be gathered from the following extract:—

"It consists in causing the patient to breathe *cool air*, while the cutaneous surface is maintained in a state of preternatural excitement, by placing him warm in bed or by enveloping his thorax with some stimulant application. I was led to the adoption of this means, by reflecting on the laws of sympathy, as inculcated by M. Broussais. That eminent physician contends, as you well know, that the sympathies between the skin, and mucous membrane of the primæ viæ are direct—that the stimulations of the one are directly repeated in the other; and he hence disapproves of the employment of blisters and other irritating applications to the abdomen, in gastritis, enteritis, &c. On the other hand, he says, that the sympathies between the skin and the mucous membrane of the lungs are reversed—whatever debilitates the former, and repels the blood from its texture, tends to irritate the latter, and produce a sanguineous congestion in the pulmonary organ, a preliminary condition of inflammation. If such be the case, one would naturally suppose that a rational mode of treating pulmonary inflammation would be to place the system, as far as possible, in a condition the reverse of that which has produced the disease. I have accordingly submitted the plan to actual trial in a number of cases in the state prison of this city, and the beneficial effects that I have uniformly obtained, render me somewhat sanguine of the value of the remedy. I do not believe that it can be solely depended upon in the treatment of acute inflammations, but, conjoined with other antiphlogistic means, it may become a powerful auxiliary. Neither do I expect it will prove a remedy for phthisis pulmonalis; for by the time the disease commonly receives this fatal appellation, the pulmonary tissue is so deeply altered and ruined, as to place the disease, in very many cases, beyond the control of medical agents; but I think it will be found to be of signal service in the early stages of these chronic inflammations. Surely, by creating a general revulsion to the external parts of the system, by means of external heat or other stimuli, and at the same time directly introducing into the inflamed lungs cold, which by its sedative properties is so efficient in allaying inflammatory irritation, and repelling the preternatural flow of blood from inflamed parts, we place the pulmonary organ in a condition well calculated to enable it to throw off the morbid action, and regain its healthy tone and functions. But facts are doubtless better than reasonings on this subject, and I will proceed to relate to you the way in which I submitted my patients to a trial of the remedy, and the results I have obtained from it.

"In order to excite and maintain the action on the surface of the body, the chest was enveloped in a vest padded with wool and lined with fur, and the patient covered warm in bed, or he was placed in a bath heated to 98°, and in this situation caused to inspire, through a tube, cool air brought from the external atmosphere, when the weather was sufficiently cold; otherwise, drawn from a reservoir in which it was cooled to about 40° of temperature, by means of ice. The operation was usually directed to be continued one hour, and repeated thrice a day; but the patients were frequently induced, from the benefit they thought they derived from it, to continue it much longer and to repeat it more frequently. They did not seem to receive any greater advantages from the warm bath than from the other mode of exciting external heat, and as its use was attended with much inconvenience, I have of late seldom employed it. The uncommon mildness of the past winter, prevented me from using air lower than 28° of temperature, which of course was raised a few degrees higher by the time it reached the mouth. None of the patients complained of such air being too cold. I found that when its temperature was not higher than 50°, it seemed to make a decided impression, and that about 40° is the temperature that answers the desired purpose very well. I have accordingly of late brought the air to that point; and, from many trials, have no doubt that from four to six quarts of ice, even in summer, will cool a sufficient quantity for a day's consumption.

"It is, I think, during the hot season, when the skin is constantly kept in an excited state, and the other secretory organs are in full play, that the remedy promises to be of more advantage in chronic inflammations than it can possibly be during the cold and variable weather of winter.

"The sensible effects produced by the remedy were tolerably uniform. When the temperature of the inspired air was not above 50°, it invariably produced an agreeable sensation of coolness in the chest, occasionally with darting pains extending to the shoulders, which the patients referred to the external and muscular parts. On persisting in the use of the remedy for a long time and repeating it frequently, they sometimes complained of a sense of soreness and fatigue in the direction of the diaphragm, and sometimes also of fulness of the head and vertigo. The most constant effect on the pulse was to render it fuller: when it was preternaturally frequent, it commonly rendered it slower; in some instances diminishing it ten to twenty pulsations in a minute; in a few cases, however, especially where it was rather slow, it rendered it somewhat more frequent. It very generally mitigated the cough, diminishing its frequency more than one-half in the course of two or three days, and rendered the expectoration freer and easier, so that the patient would frequently throw it up almost without effort. The effects on the cutaneous function were not less decided: it diminished the morbid heat, and rendered the skin more pliable and pleasant to the feel. The patients that used the remedy to any extent complained continually of great hunger, and it was with difficulty that I restrained them to a moderate allowance of vegetable food.

"Heretofore, in order to give the remedy a fair trial, I have employed it unaided by other means than the observance of low diet, and the benefits resulting have been too uniform and decided to be merely accidental. In one recent case of catarrh, it completely removed the affection in twenty-four hours. In all the old pulmonary cases it has greatly alleviated the disease; and notwithstanding the unfavourable circumstances under which these patients are placed, it still seems to be rendering them service. In one case where the cough was almost incessant, I succeeded, in a few days, in allaying it so much as to enable the patient to sleep quietly all night, and his cough is now comparatively rare and trifling. In two cases of asthma, of many years' standing, the patients acknowledged that the remedy had afforded them more relief than any other means they had employed. The fulness of the pulse, the muscular pains, and the affection of the head, appear to indicate that the blood is diverted from the thoracic organ, and accumulated in other parts of the body. If such be the case, small and repeated venesections would render the effects of the revulsion from the lungs more permanent, and materially aid in removing the chronic engorgements.

"Dr. Binsse, the resident physician of the prison, has attended to the administration of the remedy, and taken copious notes of the progress of the cases, which I shall at some future day condense for publication.

"In my next letter I will describe the refrigerator and the other parts of the apparatus, that I have contrived, for the application of this new remedial agent." —*American Journ. of the Med. Sciences.*

---

### 42. On Puerperal Convulsions. By W. Michell, Esq.

From a short chapter on this important subject, which we find in Mr. Michell's little work on the ergot of rye, we shall select some practical observations that may prove serviceable to our obstetric brethren. The investigation of puerperal convulsions has been sadly neglected,

Mr. Michell observes, in this country. "For any tolerable account of the disease, such even as any accoucheur in extensive practice could furnish, we are almost entirely indebted to foreigners." The French have given us two of the best treatises on this subject—one by Chaussier, and the other by Miguel.

"We very seldom find a true puerperal convulsion until the last months of gestation, or until the os uteri begins to dilate, or the neck to be lost in the general distension of the organ. What we observe before this period, is not puerperal convulsion, but spasmodic attacks from the pressure of the uterus on any diseased or irritable part, or from the action of the fœtus in utero on its first motion. These convulsions, or rather spasms, end in faintness, whereas the puerperal convulsions end their paroxysms with great irritability. Constant moving and shifting the situation, the carotid throbbing, stertorous breathing, with a small pulse, are the marked and dangerous termination of such paroxysms. If the attack subsides, we find after delivery, in cases of this sort, great prostration of strength. With regard to the buffy appearance of the blood in these convulsions, it is not greater than at other periods of gestation, nor even so great after the delivery has taken place. The blood assumes the buffy coat whenever any one organ is gorged with blood, to the loss of other parts of the system. We find this appearance in a very great degree in persons addicted to spirituous liquids, although there be no disease except the engorgement of the liver. In fact, in excessive circulation of any sort, attended with engorgement, we find this to be the case; but where there is no engorgement, though the circulation be quick, but not carried on with a great action, or more strictly speaking, with a strong arterial contraction, the blood does not assume this appearance. The buffy coat then, I consider, does not depend entirely on inflammation, or obstruction of the blood through any particular part, but on an excessive engorgement of a particular part, or on an imperfect circulation; otherwise we should not find, as is generally the case, that the buffy appearance does not exist in the first portion of the blood extracted on any occasion.

"From these considerations, we may learn not to be deceived by the buffy appearance of the blood in cases of convulsions. We must not attribute it to inflammation, and continue to abstract blood until the buffy coat ceases to appear, but we should rather excite the uterus to action by any means that we have in our power. While uterine action continues, the convulsions will entirely disappear. I would, therefore, earnestly recommend every practitioner not to delay the delivery until serous affusion or sanguineous apoplexy has taken place—then it will be too late to remedy the evil. I would, without hesitation, apply all the means in our power, and not leave the patient until I had been successful in my endeavours." 6.

An interesting case is related of a woman to whom Mr. Michell was called, at 3 o'clock, and whom he found held by two women.

"After a few minutes she became calm, and talked rationally, complaining of violent pain in the head, as though it were splitting. When the pains came on, she said the fits presently succeeded, and the pains ceased. In a few minutes a pain came on, as if it would be expulsive; but almost instantaneously the convulsion followed, which put an end to it. I examined and found the os uteri partially dilated, about the size of a shilling; I kept the finger applied until the pain came on, when I found the os uteri dilate for a moment; the convulsions again came on, and from the spasmodic twistings, I was unable to keep the finger applied to the os internum. I then considered it right to bleed her largely; but finding her a weak woman, I pursued a different treatment.

"After giving her antispasmodics she appeared much the same. When the convulsions subsided she appeared lost for a moment, and breathed sonorously, with extreme lassitude. At 7 o'clock I made up my mind to turn the child, as I had often found that by bringing on a right action in the uterus, the convulsions may be stopped. This I effected in a few minutes; but as soon as I dilated the os uteri, the convulsions returned in all their hideous form. I continued—dilated—and delivered the child. During the paroxysm, as I was taking away the afterbirth, the mother cried out that the child was coming, being perfectly ignorant of its birth; and even when it was shewn her, and she heard it cry, she could scarcely be induced to believe it her own child. The convulsions still continued with longer intervals. I gave her ℈1 of musk, to be doubled every paroxysm until they ceased. Three doses I have invariably found to overcome the complaint." 8.

It has been asserted, says Mr. Michell, that puerperal convulsions come on without any determination of blood to the head. This he conceives to be a mistake. On the contrary, he considers it to be one of the marked distinctions of puerperal convulsion.

"We frequently meet with convulsive spasms of the legs, from cramp, or pressure on the nerves of the lower extremities by the head of the child; but these must be distinguished from puerperal convulsion, where every muscle of the body is called into action, more particularly the heart. The action of this important muscle prevents the return of the blood, and causes a dreadful congestion on the head; and this, combined with the glassy appearance of the eye, and stertorous breathing, when preceded by the uterus acting on its orifice, I should call the marked symptoms of puerperal convulsions. Miguel speaks of convulsions in the early months of pregnancy; these I consider to be nothing more than common convulsions, not at all *dependant* on pregnancy, though perhaps *aggravated* by it; they commonly end, or wear themselves out, without the least danger to the woman; other than these, I have never met with in the early months. But I am surprised that he should call them spasms, as he calls tetanus; and should distinguish betwixt spasms and convulsions, defining spasm to be contraction without dilatation, but convulsion to consist of spasm with alternate dilatation. What, we may ask, is the cause of that action in the present case; unquestionably the pressure of the brain arising from the engorgement of its blood-vessels, as every muscle appears to be in action, excepting the one that conveyed the excitement in the first instance, and that also in many cases, but in a very imperfect manner; frequently we find in convulsions that the child has been expelled by the convulsions in a few pains, when, on other occasions, the same woman has had lingering labours, arising from the uterus acting with the other muscles; in these instances no danger is to be apprehended, as there is a sufficient quantity of blood retained by the uterus to prevent an over-distention of the vessels of the head. The name convulsion has not been given to the contraction of the heart and other parts, though strictly speaking, these are convulsions, but we find their excessive contraction designated by a term for each particular organ, as vomiting for the stomach, voiding urine for the bladder, palpitation for the heart, and hiccup for the diaphragm; but these are of little consequence to the present question.

"Once more I would impress on the accoucheur the words of Mauriceau; that convulsions are highly dangerous to both mother and child, unless the female is delivered as soon as they appear, having recourse, in certain cases, to ordinary treatment, until the os uteri begins to dilate. La Motte expresses the same opinion, but in a more equivocating manner. Gardien says you ought to wait, because labour comes on very quickly, and the child is sometimes born in a couple of pains. Two instances that I have met with support the opinion of Miguel, that convulsions are mitigated by the discharge of the Liq. Amnii. These were the only cases in which the membranes were not broken before my arrival. In one instance, the woman had no return of pain or convulsion for many hours, and the other labour proceeded very well in about twenty minutes. Miguel is of opinion that the os uteri in some cases cannot be dilated; but I have no doubt that it may be always effected by holding the woman by strength of arm. He advises the os uteri to be cut into; but this is an unnecessary and useless introduction of instruments; for in the worst cases I have seeen, the os uteri may always be dilated by a little more trouble; the only reason I can fancy for such a proceeding is, that the operator is often, in a great measure, paralysed by the shocking sight before him.

"It has often been asserted, that in some cases it is impossible to turn. I have been fortunate enough to have met with no such case. The Edinburgh Med. Mag. states, that the os uteri cannot be dilated until its neck is lost or obliterated. In general it is found that convulsions carry off the patient just as the child's head is born; this is the consequence of deferring the delivery too long. A great degree of excitement is caused by the accoucher's hand first passing through the part; and when the disease is fully formed, the moment the accoucheur assists, the fatal mischief ensues; the spasms increase, the head becomes more turgid, and, in the general struggle of nature, apoplexy is produced. Then we look back on our patient, and fruitlessly regret that we had not been more prompt in our treatment. If, then, the convulsions be not encreased to an alarming degree by the first efforts to dilate the Os Uteri, the operator should continue as quickly as possible, until it is sufficiently dilated to bring away the child; then proceed to turn. The operation of turning may in these cases be more easily effected than in any others, the Uterus being completely flaccid; if it contract at all, it is only very partially; a few fibres perhaps in one part, the other parts remaining completely flaccid, so that the child is very easily turned, and may be brought away with the greatest safety. I should advise every one, before they proceed to turn the child, to administer the Ergot of Rye. I have found it in most cases efficacious in producing expulsive efforts after the membranes have been ruptured, expelling the child generally in a few pains, and in every case it appears to remove the more alarming symptoms of the convulsions, and I therefore consider it to be highly beneficial." 12.

We have given a full account of Mr. Michell's observations on the use of the ergot of rye in difficult parturition in the Review department of this Journal. Mr. Michell is an old and experienced practitioner—consequently his remarks are highly deserving of attention.

---

### 43. Rapidly fatal Dissection Wound.

At eight o'clock in the evening of the 27th November, a young student (Mr. Kissam) cut his middle finger while examining the abdominal viscera of a dead body. He complained almost immediately of smarting pain, and quickly washed the parts clean with soap and water. He was desired by Dr. Godman, the Professor of Anatomy to suck the wound; but this he neglected, and went on with the examination, merely washing his hands when he had done. Before he got up next morning he was seized with nausea and vomiting—quick pulse—tumultuous nervous agitation. Dr. Mott ordered him to go home to his father, who was a physician; to get bled—to apply a poultice—and keep very quiet. He went home—took a purgative, but was not bled. He spent a bad day and night. On the morning of Thursday (the accident happened on Tuesday) Professor Godman met Dr. Mott and the elder Kissam in consultation. His countenance was dreadful. "The left side of his face looked very much like the face of a paralytic, the muscles seeming relaxed and powerless, although there was no distortion towards the right side. An expression of intense anxiety was imparted to the countenance, and gave an air of wildness and alarm to all his movements. He complained of great pain in his head; the arm was not remarkably painful, the wounded finger slightly so, and the wound itself exhibited nothing of an active inflammation; the pulse was not remarkably excited. The absorbents of the chest extending along the border of the pectoralis major were visible, being inflamed; none of the absorbents of the arm were similarly obvious."

Poultices—leeches—the spirit vapour-bath.* 4 *o'clock*. All the symptoms were rather aggravated than ameliorated.—There were signs of congestion about the head—the pulse full and strong. He was bled to eight ounces, with relief—great relief of all the symptoms; except the nausea and vomiting, which were increased. A blister was directed to the epigastrium—and to the insides of the legs. *Friday*. The gastric irritability continues—delirious in the night—now composed. He this morning threw up some solid fæces in the attempts to vomit. The head-ache was severe—no pain on pressure of the abdomen. Took some

---

* Dr. Godman assures us that he has often seen the most decided advantages from the spirit vapour-bath in cases of poisoned wounds.—*Ed.*

ammonia and spir. æth. comp. which he retained. He fell asleep, and remained quiet for some time. 10, P.M. The determination of blood to the head returned, and he was cupped and blistered, with considerable relief. He now, *for the first time*, complained of his finger, which was slightly swollen. Dr. G. laid open the finger freely, from the first joint to the extremity. Dr. Hosack joined in consultation, and venesection was again employed; when the violence of the symptoms was again abated. The irritability of stomach continued. At midnight Dr. Mott was called to the patient, and found him with symptoms of ileus. He administered, *per anum*, 120 drops of denarcotized laudanum. This suspended the vomiting, and induced sleep with a copious perspiration. When he awoke next morning, he was perfectly rational, and altogether so much better, that sanguine hopes of his recovery were entertained.

*Saturday*. This day all hopes ceased, as the hand of death was evidently upon him. He retained his faculties, and manifested the most perfect serenity of mind till the moment of his decease, which happened early on Sunday morning.

No examination was made; but Dr. Godman expressed his decided opinion that this young gentleman died of inflammation of the mucous membrane of the bowels—"and that the wound received in dissection was a mere coincidence, and did not produce the symptoms from which the patient suffered." We confess that, in this opinion, we cannot coincide with Dr. Godman. Inflammation of the mucous membrane of the bowels may have existed, and may have been the main cause of death; but we should be inclined to view this inflammation as a consequence of the morbid poison introduced into the system through the medium of the wound, and by no means an idiopathic and simple affection.—*American Journal of the Medical Sciences, No.* 11.

---

### 44. SIGNS OF DROWNING.

M. Orfila has made a great number of experiments with the view of determining whether submersion has taken place prior or subsequent to death—an important decision in legal medicine. The following are the inferences which he has drawn.

"That the red, livid, and swelled condition of the face, with froth at the mouth and nostrils, which some authors have laid down as indicating that the submersion has taken place during life, leads to no such inference, as it is wanting in many who have been drowned, and is present in many who have met their death by other means.

"2. That the same remarks apply to extreme paleness of the face, which is an effect of the body remaining long in the water. M. Orfila here describes the alterations which the skin undergoes in those who have been long submersed. He asserts that on the legs the integuments become indigo colour, and then brownish on exposure to the air, while the rest of the body is very white, but the moment it comes into contact with the air, it is successively converted into brown and green, commencing at the chest. Remaining long in the water also brings on abrasions of the skin, which may give rise to the idea of wounds having been inflicted.

"3. Excoriations of the fingers and traces of dirt under the nails are of no assistance, because they are wanting in those who are drowned before they come to the bottom, while they may be present in a body which, being thrown into a river, strikes against various obstacles.

"4. Injections of the brain and its membranes, M. Orfila thinks would be a satisfactory indication of drowning, if it were proved that the body became cold in a vertical position. As, however, this sign is frequently absent in those who have been drowned, so it cannot be regarded as a positive proof.

"5. In those who have been drowned, the right cavities of the heart, the venæ cavæ, the pulmonary veins and arteries, are generally distended by a quantity of black blood, while the left side of the heart and the aorta are much less filled; the right ventricle is of a blackish brown; the left of a clear rose colour; and the right cavities retain a less contractile power than the left. This condition, however, is met with in many cases of sudden death.

"6. Although the blood is generally fluid, yet M. Orfila has seen it coagulated in one individual who was drowned; a fact also observed by Lafosse, and more recently by M. Avissard.

"7. The dissection of more than fifty persons who had been drowned has satisfied M. Orfila that it is a mistake to suppose that in drowning the individuals die during inspiration, and that, in consequence, they have the diaphragm pushed into the abdomen, and the chest elevated.

"8. He regards the colour of the abdominal viscera as indicative of asphyxia in general, but not of drowning in particular.

"9. The experiments of Orfila, and some other physiologists, show that water enters the stomachs of those who drown themselves; while this is not the case with regard to bodies thrown into the water. But, in order to give its full value to this sign, it would require to be proved that the water had neither been swallowed before death, nor injected after it.

"10. It is not true that the epiglottis is pushed down upon the larynx.

"11. Great importance had been attributed to the presence of sanguineous froth in the windpipe. M. Orfila objects, however, that this exists in other cases, as in death from epilepsy and hanging; while it is wanting in the drowned who have remained long under water, without coming to the surface to breathe.

"M. Orfila made a considerable number of experiments to ascertain if water enters into the ramifications of the bronchia of the drowned; and he has always found that the fluid entered the lungs of the animal, and that the quantity was greater or less in proportion to the care took to keep the head erect in lifting the animal out of the water. But the fluid is not found in the air-passages, if the examination be made soon after death. As to the question, whether the water enters the lungs after death, the experiments of M. Orfila agree with those of M. Piorry, proving that it does enter, and more completely the more vertical the position of the animal; and consequently, the presence of water is not a sufficient proof of drowning, as water enters the air-passages of the dead body, and that the only absolute sign is the presence of water in the minute ramifications of the bronchia; it being proved that this water is the same as that in which the individual was immersed, that it had been injected after death, and could not have penetrated to the lungs from the vertical position of the body in the liquid."

---

### HOSPITAL PRACTICE.

### 45. ABSCESS IN THE LUNGS AFTER INJURIES AND OPERATIONS.

[St. George's Hospital.]

Prior to the publication of Mr. Rose's paper in the Medico-Chirurgical Transactions, the attention of the profession in this country was but little directed to the above very fatal affection. It is true that Mr. Guthrie and Mr. Bell had previously attended to it, and that its frequency and importance had been appreciated and perhaps overrated by Dessault upon the Continent. These notices, however, were diffused in elaborate works, and quite beyond the reach of the majority of those, who become acquainted by means of the medical journals, with the transactions of medical societies. Mr. Rose's paper, or an abstract of it, will therefore be read where the works of Mr. Bell or Mr. Guthrie most probably will not, and with the view of contributing some further illustrations, we intend to bring together some cases which have recently occurred.

We alluded, in a previous article, to the frequent, we might almost say constant, occurrence of pulmonary abscesses or pleural inflammation, as a sequence or consequence of phlebitis. Cavil as we may on the why and the wherefore, the fact is undeniable, and as we have dwelt upon the subject already, we pursue it no further at present.

The case which we are going to detail occurred very recently at St. George's, and, as usually happens, the affection of
